# Progress in Surgery

**Published:** 1899-05-06

**Authors:** 


### Progress in Surgery.

### ORTHOPÆDIC SURGERY.

**Coxa Vara.**—Royal Whitman has made some further observations on coxa vara[1] founded on the examination of 26 cases during the last four years. It is one of the group of deformities of the lower extremity due to inherent or acquired weakness of structure, or to disproportionate strain, or both. The disability is often out of all proportion to the apparent deformity. There is relative prominence and elevation of the trochanters, and owing to depression of the neck of the femur backwards the normal lumbar lordosis is diminished. Whitman remarks that in the majority of his cases there is no evidence of general rhachitis. It may be distinguished from congenital dislocation by flexing and adducting the limb, when, in the latter, the head and neck of the bone can be made out, but not in the former. As regards the ætiology, although the affection usually occurs in adolescence, a latent deformity acquired in childhood may be a factor of considerable importance; thus rickets may be an indirect cause. Concerning operative treatment, Whitman has performed cuneiform section of the base of the great trochanter in three cases, and linear osteotomy below the trochanter minor in two cases. Of the 26 cases 20 were in males and 6 in females, and the condition was bilateral in 5 only.

Watson Cheyne, in describing the result of division of the femur for coxa vara, confirms the opinion of Whitman that the progress of the deformity in the neck of the bone may be arrested by the operation.[2] He found that in the case of the leg not operated on there was progressive raising of the level of the great trochanter as age advanced. Cheyne suggests that this effect may be due to sclerosis of the softened neck set up by the operation. Royal Whitman seems to regard it as being due to the alteration in the inclination of the neck of the femur. He evidences the fact in support of his argument that after fracture of the neck of the femur in young children the neck of the femur is apt to bend causing coxa vara, which he regards as being due to the mechanical disadvantage of the altered angle. Another reason given by Cheyne for the ultimate comparative lengthening of the limb operated upon is that the nutrient artery to the neck of the bone may be divided in the operation, with diminished vascularity and consequent consolidation of the neck.

**Pott's Disease.**—Sufficient time has now elapsed to enable some opinion to be formed of the value of Calot's method of forcibly straightening the spine. Gevaert comes to the following conclusions[3]: (1) Calot's method undoubtedly has dangers, and at least fourteen deaths have already been reported as directly due to it. (2) Chloroform is always a danger in the operation and is best avoided. (3) Reduction is made easy by various forms of mechanical contrivances which exert traction and pressure with extension. (4) Bandages on the head, neck, and shoulders are very uncomfortable and apt to produce ulcers. (5) The diagnosis of the possibility of reduction can be made only by attempting it to determine the presence or absence of ankylosis. Great force should not be employed. (6) Persistent paraplegia is an indication for reduction. (7) Radiographs furnish valuable information relative to possibilities of treatment. (8) Most authors repudiate laminectomy and other open operations. (9) Pott's disease is cured in two ways—(*a*) by ankylosis of the bodies of the vertebræ, (*b*) by ankylosis of the neural arches. It is therefore absolutely essential to respect the posterior arches, and not to weaken them by osseous resection or ligamentous excisions. (10) Calot's method should not be attempted in weakly children, in those with cough, abscesses, fistulæ, or visceral complications.

The operation has been performed several times by Goldthwait, who says that no unpleasant results have been experienced, and that in five cases in which paralysis was present the recovery was almost immediate. Wullstein cites the experimental results in the cadaver as contra-indications for immediate correction of deformity. These include laceration of the pleura, mediastinal hæmorrhages, ruptured abscesses, laceration of the dura mater, and torsion of the spinal cord. Accordingly Wullstein has devised apparatus for the gradual extension of the spine with the patient in a supine position, the chest being unrestrained. It consists essentially of an iron frame acting as a stretcher, with extension applied to the shoulders and legs. It is now found that in the correction of the deformity in early cases an anæsthetic is not necessary, the extension being performed by the body-weight alone, with the patient supine, and a plaster jacket is straightway applied. In this way no complications seem to arise, and in five cases immediate improvement in the paralysis occurred. Goldthwait states that this experience would seem to show that, in spite of the varying theories as to the cause of the paralysis, pressure must be the most important element, and that there can be little degeneration, for in some cases the improved motor control has been immediate.

**Lateral Curvature of the Spine.**—Much has been written of late by Richard Barwell on "amesial pelvis" associated with scoliosis, which, he says, was unsuspected until he mentioned the subject in 1895. The condition is sometimes so obvious that this remark is scarcely credible. Barwell assigns amesiality of pelvis as a cause of spinal curvature, and not as an effect or concomitant, as most surgeons would believe. He says amesial pelvis is of necessity followed by lateral curvature of the spine, and that pelvic aberrations are most important factors in the production of the deformity, and their correction most essential to treatment. Amesiality of pelvis he defines as a twist or rotation of the pelvis on a vertical axis, so that one side of the bone lies further back than the other. The malposition is present during sitting as well as standing, and the origin of the trouble is frequently a faulty position in sitting, chiefly when the child is learning to write.

There are several varieties of torsion of the pelvis which require consideration. (*a*) Permanent obliquity, when one iliac crest is lower than the other. In these cases one limb may be found shorter than the other. (*b*) Amesiality: This is usually due to faulty sitting, and is detected by holding up a plumb-line and seeing if the vertebra prominens and the spaces between the nates and the malleoli are in that line. (*c*) Version: In this one buttock is on a posterior plane to the other. This is detected by looking down the back from above.

The reader will observe from the above that it is simply a resolution of the usual pelvic deformity into the component deviations, Now every alteration in the position of the pelvis necessitates a compensating change in the spine above. A habitual malposition below will necessitate a habitual compensation above, and when use becomes second nature the deformity becomes fixed. By preventing the pelvic malposition we may therefore prevent the scoliosis.

In the treatment of these conditions the causes of pelvic obliquity must first be remedied. Such are shortness of one limb or ovarian neuralgia, causing the advance of one foot. Amesiality must be treated by postural exercises and educating the patient into the appreciation of a normal neuro-muscular position. Version must be treated by correcting the position in sitting, and by postural exercises against a wall in standing.

[1] New York Med. Jour., Jan. 21, 1899. [2] Brit. Med. Jour., Feb. 18, 1899. [3] Amer. Jour. Med. Sci., Feb., 1899. [4] Brit. Med. Jour., Jan. 21, and Feb. 4, 1899.

